# A human iPSC-derived inducible neuronal model of Niemann-Pick disease, type C1

**DOI:** 10.1186/s12915-021-01133-x

**Published:** 2021-10-01

**Authors:** Anika V. Prabhu, Insung Kang, Raffaella De Pace, Christopher A. Wassif, Hideji Fujiwara, Pamela Kell, Xuntian Jiang, Daniel S. Ory, Juan S. Bonifacino, Michael E. Ward, Forbes D. Porter

**Affiliations:** 1 Division of Translational Medicine, Eunice Kennedy Shriver National Institute of Child Health and Human Development, National Institutes of Health, DHHS, 10CRC, Rm. 5-2571, 10 Center Dr, Bethesda, MD USA; 2 Neurosciences and Cellular and Structural Biology Division, Eunice Kennedy Shriver National Institute of Child Health and Human Development, National Institutes of Health, DHHS, Bethesda, MD 20892 USA; 3 Department of Medicine, Washington University School of Medicine, St. Louis, MO 63110 USA; 4 Diabetic Cardiovascular Disease Center, Washington University School of Medicine, St. Louis, MO 63110 USA; 5 National Institute of Neurological Disorders and Stroke, National Institutes of Health, DHHS, Bethesda, MD 20892 USA

**Keywords:** Human induced pluripotent stem cells, Human neurons, Niemann-Pick disease, type C1, Neurodegeneration, NPC1, Lysosomal disease

## Abstract

**Background:**

Niemann-Pick disease, type C (NPC) is a childhood-onset, lethal, neurodegenerative disorder caused by autosomal recessive mutations in the genes *NPC1* or *NPC2* and characterized by impaired cholesterol homeostasis, a lipid essential for cellular function. Cellular cholesterol levels are tightly regulated, and mutations in either *NPC1* or *NPC2* lead to deficient transport and accumulation of unesterified cholesterol in the late endosome/lysosome compartment, and progressive neurodegeneration in affected individuals. Previous cell-based studies to understand the NPC cellular pathophysiology and screen for therapeutic agents have mainly used patient fibroblasts. However, these do not allow modeling the neurodegenerative aspect of NPC disease, highlighting the need for an in vitro system that permits understanding the cellular mechanisms underlying neuronal loss and identifying appropriate therapies. This study reports the development of a novel human iPSC-derived, inducible neuronal model of Niemann-Pick disease, type C1 (NPC1).

**Results:**

We generated a null i3Neuron (inducible × integrated × isogenic) (*NPC1*^*−/−*^ i^3^Neuron) iPSC-derived neuron model of NPC1. The *NPC1*^*−/−*^ and the corresponding isogenic *NPC1*^*+/+*^ i^3^Neuron cell lines were used to efficiently generate homogenous, synchronized neurons that can be used in high-throughput screens. *NPC1*^*−/−*^ i^3^Neurons recapitulate cardinal cellular NPC1 pathological features including perinuclear endolysosomal storage of unesterified cholesterol, accumulation of GM2 and GM3 gangliosides, mitochondrial dysfunction, and impaired axonal lysosomal transport. Cholesterol storage, mitochondrial dysfunction, and axonal trafficking defects can be ameliorated by treatment with 2-hydroxypropyl-β-cyclodextrin, a drug that has shown efficacy in NPC1 preclinical models and in a phase 1/2a trial.

**Conclusion:**

Our data demonstrate the utility of this new cell line in high-throughput drug/chemical screens to identify potential therapeutic agents. The *NPC1*^*−/−*^ i^3^Neuron line will also be a valuable tool for the NPC1 research community to explore the pathological mechanisms contributing to neuronal degeneration.

**Graphical abstract:**

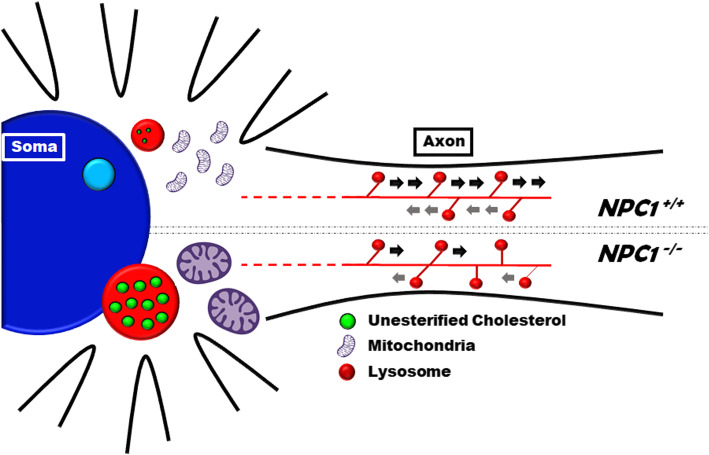

**Supplementary Information:**

The online version contains supplementary material available at 10.1186/s12915-021-01133-x.

## Background

Cholesterol is an essential molecule; however, its cellular levels must be carefully balanced. Various inborn errors of cholesterol homeostasis result in excessive or insufficient cellular cholesterol, which can lead to severe mental and physical abnormalities. One such example is Niemann-Pick disease, type C (NPC). NPC is an autosomal recessive disease, characterized by the accumulation of unesterified cholesterol in the late endosome/lysosome compartment and progressive neurodegeneration in affected individuals [1]. NPC disease is the result of mutations in either *NPC1* or *NPC2*. The proteins encoded by these genes play a sequential role in the efflux of cholesterol from the late endosomal/lysosomal compartment [2]. Numerous cell-based studies of NPC1 have utilized patient fibroblasts for studying cellular pathology and conducting high-throughput drug/compound screens [3]. However, from a clinical standpoint, progressive neurodegeneration is the most salient clinical aspect. Thus, it is critical to identify and understand the cellular processes that contribute to neuronal loss and to identify therapies that are effective in neurons.

The i^3^Neuron (inducible × integrated × isogenic) platform is a scalable iPSC-derived neuron technology that allows for the reliable and reproducible generation of highly pure human neurons in vitro [4]. The i^3^Neurons are a recently developed human induced pluripotent cell (iPSC) with a doxycycline-inducible neurogenin 2 (NGN2) transgene integrated into the AAVS1 locus (Figure S1A). Upon exposure to doxycycline, the iPSCs synchronously differentiate into a homogeneous population of glutamatergic neurons within 10 days (Figures S1B and S1C). This scalable, rapid, two-step protocol is well suited to generate large numbers of relatively homogeneous NPC1 null (*NPC1*^*−/−*^) and isogenic control (*NPC1*^*+/+*^) neurons, which can be used for molecular, biochemical, and cellular experiments. These characteristics also make the *NPC1*^*−/−*^ i^3^Nerurons attractive for use in genome-wide genetic and large-scale high-throughput drug screens. This contrasts with traditional iPSC-derived neurons that require a more complex and slower differentiation process that yields a less homogeneous cell population after neuronal differentiation.

In this study, we describe the development and characterization of an *NPC1*^*−/−*^ i^3^Neuronal line. *NPC1*^*−/−*^ i^3^Neurons manifest the expected NPC1 cellular pathology of increased storage of unesterified cholesterol in the endolysosomal compartment and formation of multi-laminar storage bodies, replicate the expected lysosomal and mitochondrial dysfunction, and have impaired axonal lysosomal transport. The mitochondrial and axonal trafficking defects can be ameliorated by treatment with 2-hydroxypropyl-β-cyclodextrin. 2-Hydroxypropyl-β-cyclodextrin has been shown to be effective in preclinical mouse [5, 6] and cat [7] models of NPC1 and appeared to have clinical efficacy in a phase 1/2a trial [8], thus providing a proof of principle that this unique model may have significant utility in high-throughput screens to identify potential therapeutic agents.

## Results

### Generation of *NPC1*^*−/−*^ i^3^Neuronal lines and characterization of the cellular phenotype

**Fig. 1:**
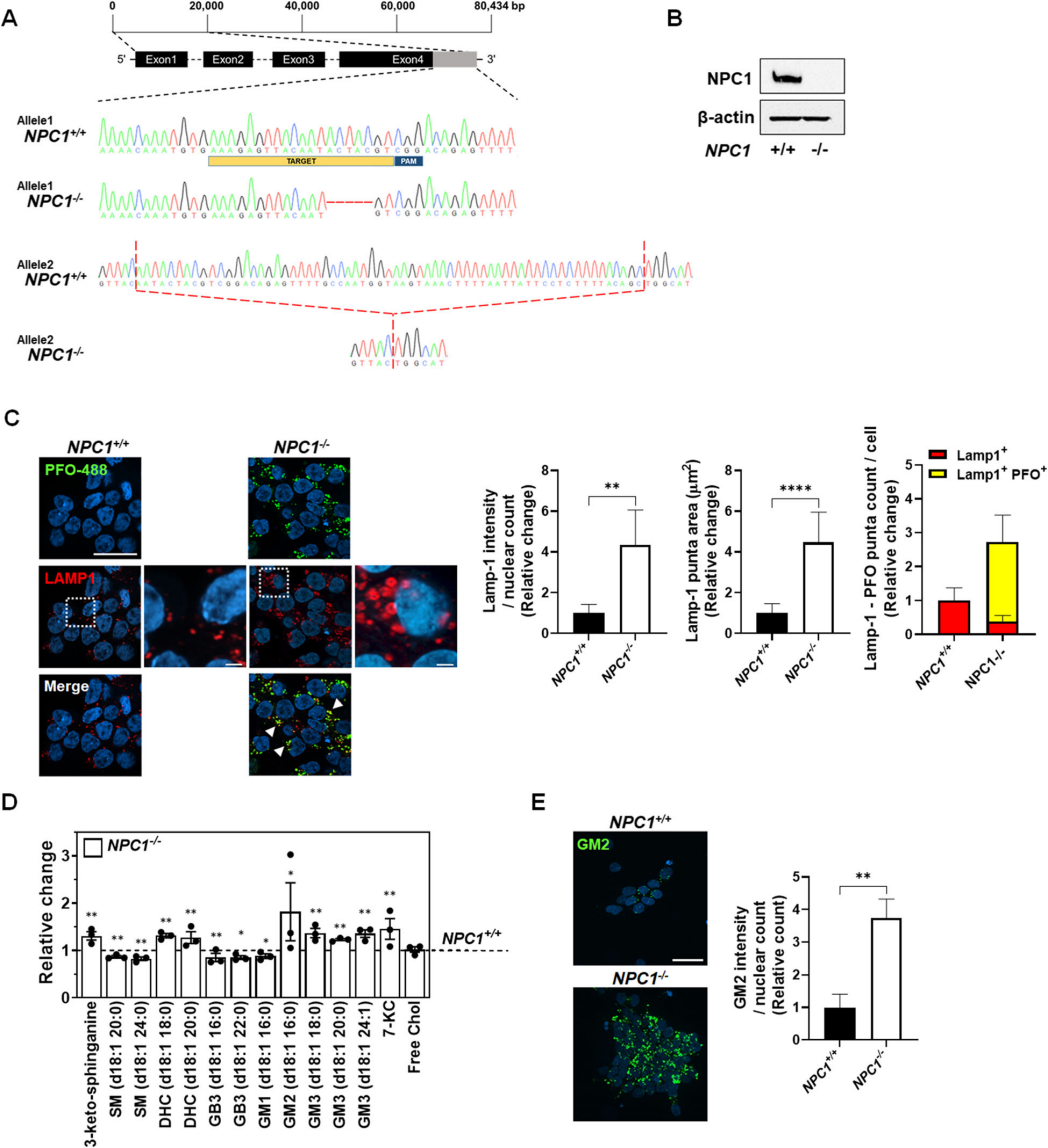
Generation of an *NPC1* mutant i^3^Neuron cell line. **A** The genomic structure of *NPC1*, reference DNA sequence, and targeting guide RNA are shown in the top part of this panel. Sanger sequencing of *NPC1*^*−/−*^ i^3^Neuron clones identified two independent deletions in exon 4 of NPC1. Allele 1 is a 5-bp exonic deletion (c.437-441del p.Tyr146fsX167), and allele 2 is a 64-bp deletion (c.434_463+36del) extending from exon 4 to intron 4. A minimum of 4 clones corresponding to each allele were sequenced, and no other *NPC1* mutations were detected. **B** Protein levels of NPC1 and β-actin in *NPC1*^*+/+*^ and *NPC1*^*−/−*^ i^3^Neurons were analyzed using Western blotting. Blots are representative of three independent experiments. **C ***NPC1*^*+/+*^ and *NPC1*^*−/−*^ i^3^Neurons were differentiated for 10 days, and endolysosomal accumulation of unesterified cholesterol was visualized by staining with PFO-488 (green) and anti-LAMP1 (red). The nuclei were counter-stained with Hoechst (blue). Data are from 30 cells per condition in average: representative of 5 independent experiments (*n* = 5). Lamp1 area of puncta counts was calculated from *n* ≥ 10 cells and averaged per cell. Scale bar, 10 μm. Magnified inset scale bar, 1 μm. **D** Lipid levels measured in *NPC1*^*+/+*^ and *NPC1*^*−/−*^ i^3^Neurons by LC-MS. Data is plotted relative to *NPC1*^*+/+*^ i^3^Neuron levels. Dotted line represents an *NPC1*^*−/−*^*/NPC1*^*+/+*^ ratio of 1. Individual points represent independent measurements, and one independent experiment is a representative of three samples for each group. **E ***NPC1*^*+/+*^ and *NPC1*^*−/−*^ i^3^Neurons were stained with anti-GM2 ganglioside (green) and Hoechst nuclear stain (blue) 10 days post-differentiation. Data are from at least 500 cells per condition, representative of 3 independent experiments (*n* = 3). Scale bar, 10 μm. *p < 0.05, **p < 0.01, ****p < 0.0001 by Mann-Whitney test when comparing two independent samples

To generate an i^3^Neuron model of NPC1 disease, we used CRISPR/Cas9 genomic editing to disrupt the NPC1 gene in CRISPRi-i^3^Neurons [4, 9, 10]. Two sgRNAs, 5′-CCTACTGAACCTGTTTTGTGAGC-3′ and 5′-AAAGAGTTACAATACTACGTCGG-3′ (PAM sequences underlined), were used to target exon 4 of *NPC1*. Only the second sgRNA successfully targeted NPC1. Multiple clones were identified that manifested the classical NPC unesterified cholesterol storage phenotype. Subsequent analyses used a compound heterozygous line with a 5-bp deletion c.437-441del (p.Tyr146fsX167) in one allele and a 64-bp deletion c.434_463+36del in the second allele (*NPC1*^*−/−*^; Fig. 1A). Western blot analysis confirmed undetectable levels of the NPC1 protein (Fig. 1B) in the *NPC1*^*−/−*^ i^3^Neurons, thus confirming a null genotype. This *NPC1*^*−/−*^ cell line had a normal karyotype (Figure S2A), and neuronal differentiation was indistinguishable from the isogenic control line (Figure S2B). NPC1 is characterized by the accumulation of unesterified cholesterol in the acidic cellular compartments [1], which can be visualized by staining with the fluorescently labeled cholesterol-binding agent perfringolysin-O (PFO) [11]. As anticipated, the *NPC1*^*−/−*^ i^3^Neurons showed increased PFO staining (Fig. 1C). Appearing as distinct puncta, the PFO signal co-localized with lysosomal-associated membrane protein 1 (LAMP1), indicating that cholesterol accumulation is occurring in the endo-lysosomal system (Fig. 1C). The LAMP1-positive organelles showed increased signal intensity and size in the *NPC1*^*−/−*^ i^3^Neurons, consistent with increased volume of the endolysosomal compartment. Qualitatively, the LAMP1-positive organelles also appear to accumulate in the soma of *NPC1*^*−/−*^ i^3^Neurons, which may indicate that the lysosomes are less mobile and remain in the perinuclear region (Fig. 1C).

### Lipidomic analysis

Accumulation of glycosphingolipids in neurons is a predominant cellular finding in NPC1 [12, 13]; thus, we performed lipidomics to obtain a comprehensive profile of the changes in lipid levels in *NPC1*^*−/−*^ i^3^Neurons (Fig. 1D). Notably, this analysis identified increases in GM2, GM3, 3-keto-sphinganine, 7-keto-cholesterol, and dihydroceramides in *NPC1*^*−/−*^ relative to *NPC1*^*+/+*^ i^3^Neurons (Fig. 1D). Increased GM2 accumulation was also demonstrated by immunohistochemistry (Fig. 1E). Decreased levels of sphingomyelin, globotriaosylceramide, and GM1 were observed in the NPC1 mutant i^3^Neurons. This lipid profile is consistent with what has been previously shown in the NPC1 mouse models and human NPC1 brain tissue [12, 14].

### Investigation of mitochondrial and lysosomal dysfunction

**Fig. 2:**
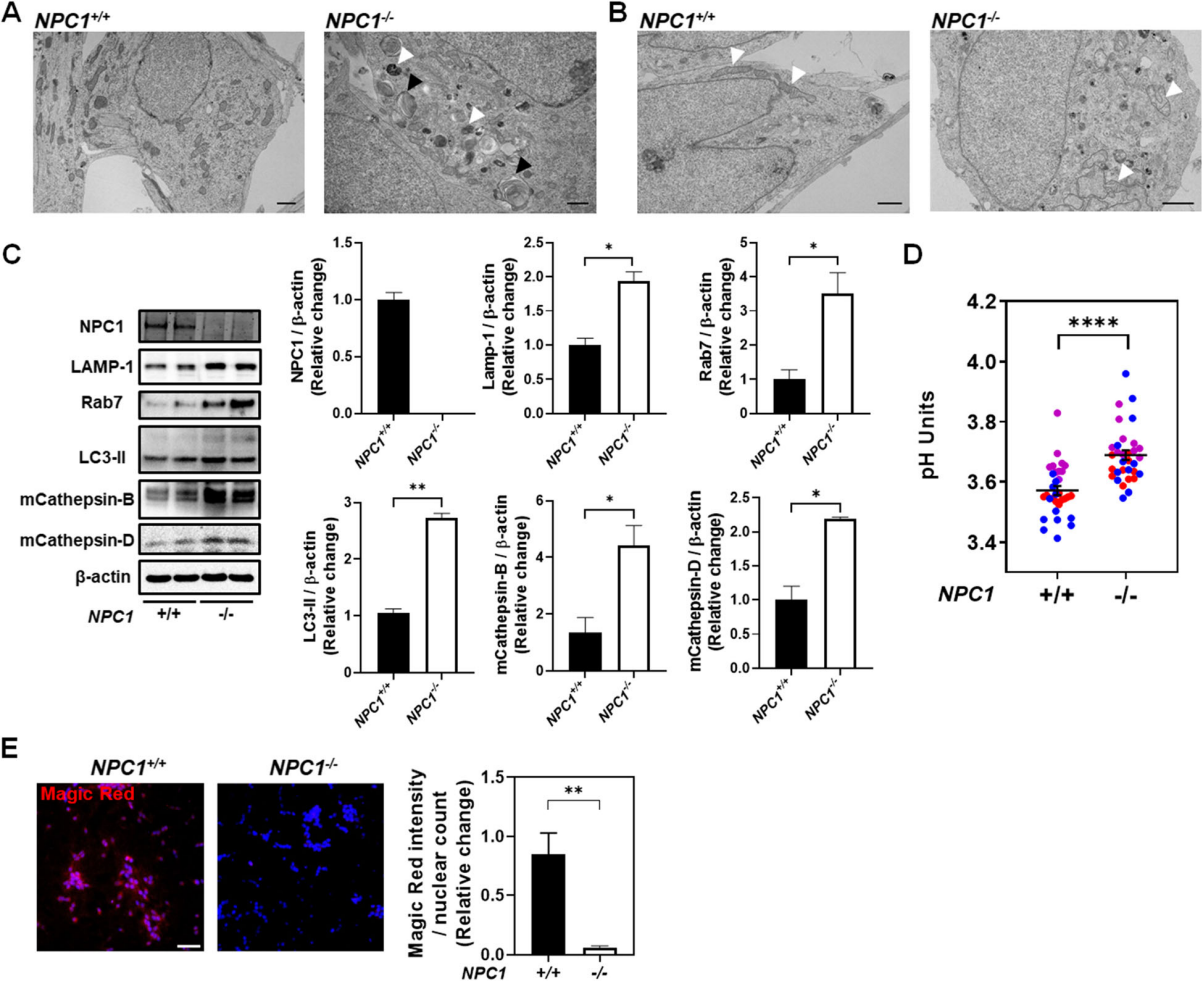
Mutant NPC1 i^3^Neurons exhibit lysosomal dysfunction. Electron microscopy of *NPC1*^*+/+*^ i^3^Neurons (**A**) and *NPC1*^*−/−*^ i^3^Neurons (**B**) was performed at 21 days post-differentiation. **A** Electron-dense structures (lysosomes) and characteristic lamellar bodies (lysosomal storage) are indicated by white and black arrowheads, respectively. Scale bar, 1 μm. **B** Mitochondria are indicated by white arrowheads in both *NPC1*^*+/+*^- and *NPC1*^*−/−*^-differentiated neurons. Images are representative of at least three independent experiments. Scale bar, 1 μm. **C** Protein levels in *NPC1*^*+/+*^ and *NPC1*^*−/−*^ i^3^Neurons 10 days post-differentiation were analyzed by western blot. The presented data is representative of three independent experiments (*n* = 3). **D** Lysosomal pH was measured in *NPC1*^*+/+*^ and *NPC1*^*−/−*^ i^3^Neurons using dual wavelength ratio imaging. Each point represents an independent measurement, and three independent experiments are displayed using different colors. **E** *NPC1*^*+/+*^ and *NPC1*^*−/−*^ i^3^Neurons were differentiated for 10 days, fixed, and stained with Magic Red (red) to assess the cathepsin B function and Hoechst (blue, nuclei). Magic Red signal intensity was plotted relative to nuclear count. In average, 100 cells were counted. The results were obtained from four independent experiments (*n* = 4). Scale bar, 50 μm. *p < 0.05, **p < 0.01, ****p < 0.0001 using Mann-Whitney test

Electron microscopy of the *NPC1*^*−/−*^ i^3^Neurons showed accumulation of dark, electron-dense bodies consistent with lysosomes and large multi-lamellar structures (Fig. 2A). Multi-lamellar inclusion bodies are characteristic of NPC1 [15]. Rat neurons with similar multi-lamellar bodies were shown to be a site of unesterified cholesterol accumulation [16]. The *NPC1*^*−/−*^ i^3^Neurons also possess numerous aberrantly shaped mitochondria (recognizable by the appearance of mitochondrial cristae) relative to *NPC1*^*+/+*^ i^3^Neurons (Fig. 2B).

Expression levels of endolysosomal proteins by western blot identified significant increases in LAMP1, Rab7, LC3-II, and the mature forms of cathepsins B and D (Fig. 2C). To assess the lysosomal function, we measured the lysosomal pH of *NPC1*^*+/+*^ and *NPC1*^*−/−*^ i^3^Neurons using pH-dependent dextrans [17]. Differentiated *NPC1*^*+/+*^ and *NPC1*^*−/−*^ i^3^Neurons were exposed to pH-dependent dextrans for 12 h. This allowed sufficient time for the pH-dependent dextrans to be endocytosed and delivered to the lysosomal compartment. These experiments showed a significant lysosomal pH increase in the *NPC1*^*−/−*^ i^3^Neurons of 0.2 pH units relative to control i^3^Neurons (Fig. 2D); similar results have been noted by Wheeler et al. [18]. An acidic environment is essential for proper lysosomal function; thus, to determine if this pH increase had a functional consequence, we measured cathepsin B enzymatic activity. Although the level of cathepsin B protein was increased, a cathepsin B cleavage assay revealed that the enzymatic activity of cathepsin B was significantly decreased in *NPC1*^*−/−*^ i^3^Neurons (Fig. 2E). Taken together, these data indicate that the *NPC1*^*−/−*^ i^3^Neuron lysosomes are both morphologically and functionally abnormal.

### Cellular response to 2-hydroxypropyl-β-cyclodextrin treatment

**Fig. 3:**
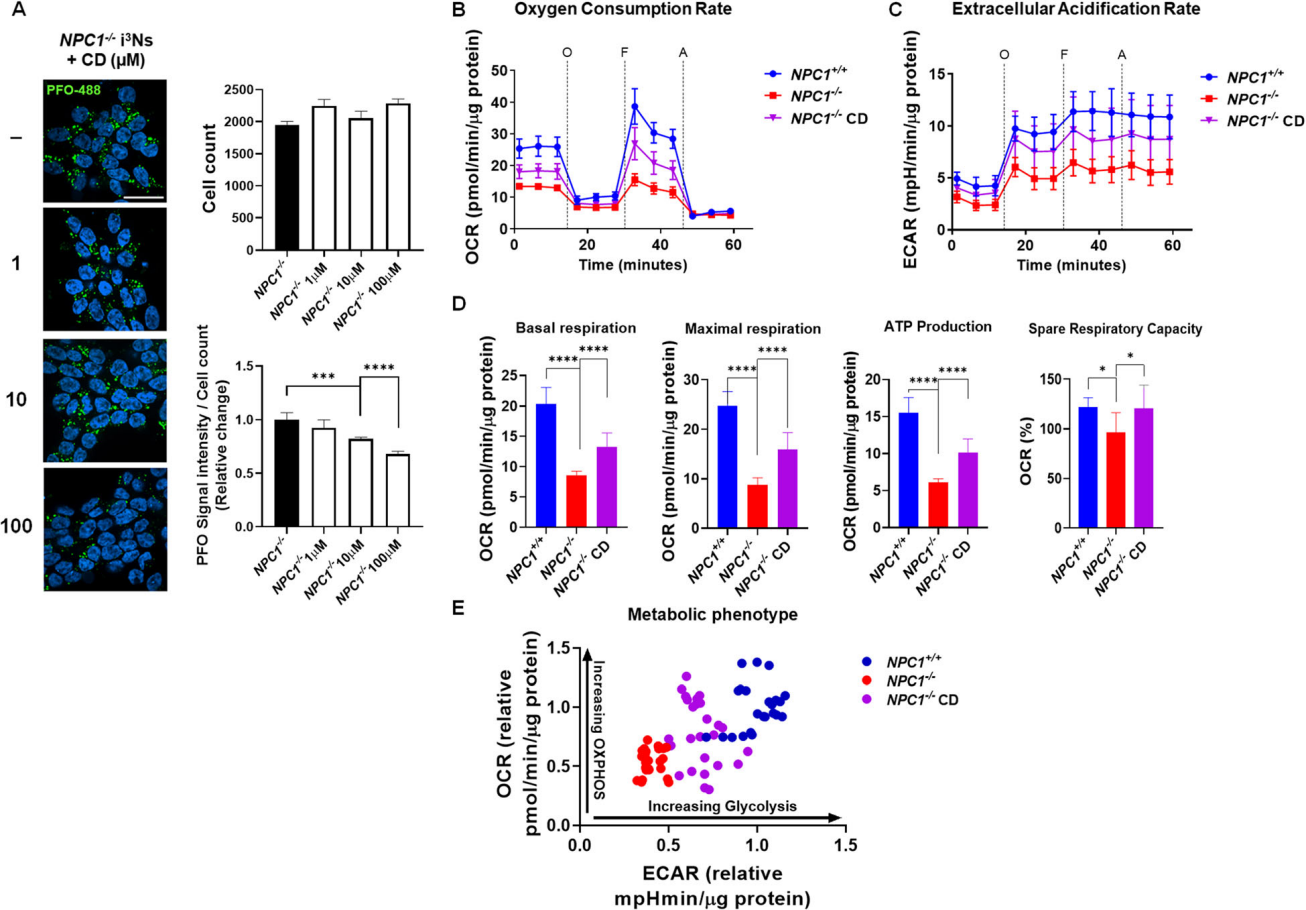
HPβCD reduces unesterified cholesterol accumulation and improves mitochondrial function in *NPC1*^*−/−*^ i^3^Neurons. **A** Unesterified cholesterol accumulation was decreased by treatment with HPβCD. *NPC1*^*−/−*^ i^3^Neurons were differentiated for 10 days and treated with 0–1000 μM hydroxypropyl-β-cyclodextrin (HPβCD) for 24 h. Cells were fixed and stained with PFO-488 (green). The nuclei were counter-stained with Hoechst (blue). At least 2000 cells were counted, and the results were obtained from five independent experiments (*n* = 5). Scale bar, 10 μm. **B**–**E** *NPC1*^*−/−*^ i3Neurons have impaired mitochondrial function that is partially corrected by treatment with HPβCD. *NPC1*^*+/+*^ and *NPC1*^*−/−*^ i^3^Neurons were differentiated for 3 days and seeded in a Seahorse XF96 plate and differentiated for another 7 days. NPC1 i^3^Neurons were treated with and without 100 μM HPBCD (CD) for 24 h prior to analysis with the Seahorse XF Analyzer to measure the oxygen consumption rate (**B**); extracellular acidification rate (**C**); basal respiration, maximal respiration, ATP production, and spare respiration capacity (**D**); and energy phenotype profile (**E**). Individual inhibitors oligomycin A (O), FCCP (carbonyl cyanide-4-trifluoromethoxyphenylhydrazone) (F), Ant A-Rot (A) are added in each time point (*n* = 8 for each group). *p < 0.05, **p < 0.01, ****p < 0.0001 by non-parametric one-way ANOVA

2-Hydroxypropyl-β-cyclodextrin (HPβCD) has been shown to decrease unesterified cholesterol accumulation in *NPC1* mutant fibroblasts [6]. In vivo studies have shown that HPβCD can decrease unesterified cholesterol storage in NPC1 zebrafish [19], mouse [5, 20], and cat [7] models. Furthermore, treatment with HPβCD has been shown to reduce neurological signs and increase survival in both the mouse and cat models. A phase 1/2a study demonstrated the potential clinical efficacy of HPβCD (Kleptose HPB, VTS-270) in NPC1 trial participants [8]. We thus investigated whether HPβCD (Kleptose HPB) would decrease unesterified cholesterol accumulation in *NPC1*^*−/−*^ i^3^Neurons. Figure 3A shows that HPBCD reduced PFO staining in a dose-responsive manner in *NPC1*^*−/−*^ i^3^Neurons. To further demonstrate the potential utility of this cell model for drug screening, we evaluated three different cyclodextrins (HPβCD, sulfobutylether-β-cyclodextrin, and sulfobuylether-γ-cyclodextrin). Consistent with what has been reported in the *Npc1* mutant mouse model (Davidson et al. 2016), HPβCD appeared to be the most effective (Figure S3). These experiments provide a proof of principle that the *NPC1*^*−/−*^ i^3^Neurons may have utility in high-throughput drug screens.

### Characterization of mitochondrial function

Given the ultrastructural abnormalities of the mitochondria in *NPC1*^*−/−*^ i^3^Neurons (Fig. 2B), we evaluated the mitochondrial function. Oxygen consumption rate (OCR) of differentiated i^3^Neurons was measured using a Seahorse XFe96 analyzer [21]. Basal and maximal OCR were decreased in *NPC1*^*−/−*^ i^3^Neurons compared to *NPC1*^*+/+*^ i^3^Neurons (Fig. 3B).

The deficit in energy production in *NPC1*^*−/−*^ i^3^Neurons was also observed in the extracellular acidification rate (ECAR) (Fig. 3C), a complementary measurement of the cellular aerobic glycolysis rate. Treatment of the *NPC1*^*−/−*^ i^3^Neurons with 100 μM HPβCD partially corrected the mitochondrial functional deficit and increased both OCR and ECAR toward control levels (Fig. 3B, C). Basal respiration, maximal respiration, ATP production, and spare respiratory capacity demonstrated a significant decrease in *NPC1*^*−/−*^ i^3^Neurons which increased toward control values after exposure to 100 μM HPβCD (Fig. 3D). The OCR and ECAR data are consistent with impaired glycolysis and oxidative phosphorylation in *NPC1*^*−/−*^ i^3^Neurons which is partially corrected by treatment with HPβCD (Fig. 3E).

### Lysosomal axonal transport

**Fig. 4:**
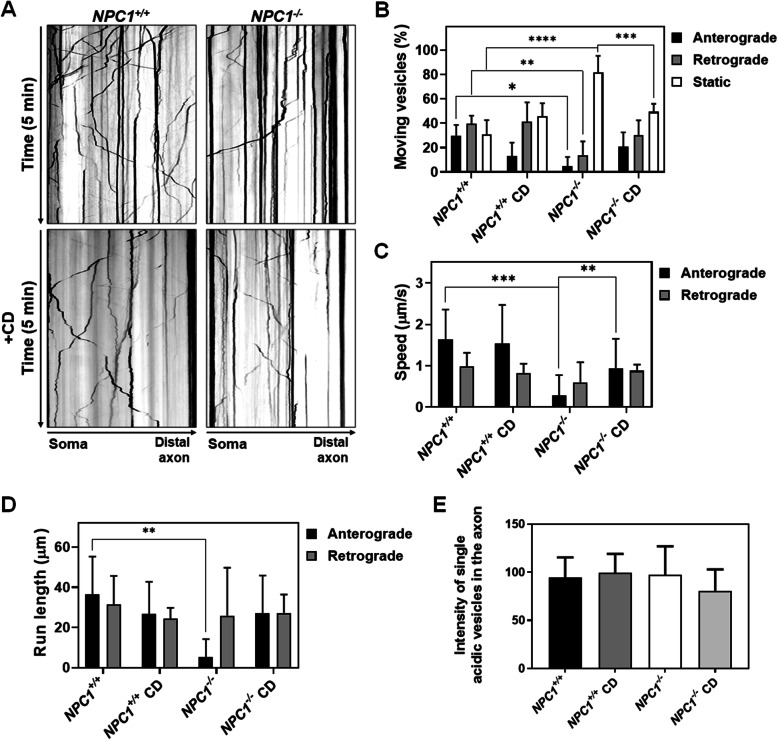
Axonal transport of acidic vesicles is impaired in *NPC1*^*−/−*^ i^3^Neurons and can be improved with HPβCD treatment. **A** Kymograph analysis of acidic vesicle movement in axons of *NPC1*^*+/+*^ and *NPC1*^*−/−*^ i^3^Neurons shows decreased movement in NPC1 mutant neurons. Images are represented in inverted grayscale. Lines with negative or positive slopes in the kymographs correspond to lysosomes moving in the anterograde or retrograde direction, respectively. Vertical lines correspond to static lysosomes. Quantification of percent and direction of moving acidic vesicles (**B**), speed of movement (**C**), and run length (**D**). All three parameters are impaired in axons of *NPC1*^*−/−*^ i3Neurons and improve by treatment with 100 μM HPβCD (CD). **E** The average intensity of single lysosomes in the axon showed no significant differences. Values are the mean ± SD from > 100 vesicles in six neurons per condition. *p < 0.05, **p < 0.01, ***p < 0.001, ****p < 0.0001 by non-parametric one-way ANOVA

The accumulation of lysosomes in the soma of the *NPC1*^*−/−*^ i^3^Neurons (Figs. 1C, 3A) suggested that lysosome transport from the soma to the distal axon might be impaired. To test this, we cultured i^3^Neurons in a grooved micro-chamber that promotes parallel axonal extension in a manner that is amendable to visualization of axonal transport. Differentiated neurons were stained with LysoTracker Red. LysoTracker Red is a vital dye that stains acidic organelles. Using time-lapse imaging at high acquisition frequency for 5 min. we were able to characterize acidic vesical transport in control and NPC1 mutant neurons. Kymographs showing movement of LysoTracker Red-positive vesicles are shown in Fig. 4A. The kymographs clearly show the decreased axonal movement of lysosomes in mutant neurons relative to control i^3^Neurons. Quantification of axonal acidic vesicle transport showed that both retrograde (toward the cell body) and anterograde (away from the cell body) movements were significantly decreased in the *NPC1*^*−/−*^ i^3^Neurons (Fig. 4B). We also observed that a large number of acidic vesicles appeared stationary in the NPC1 mutant axons during the observation period. Specifically, 69.3 ± 10.7% of the LysoTracker Red-positive vesicles were motile in the *NPC1*^*+/+*^ versus 18.6 ± 12.6% in the *NPC1*^*−/−*^ i^3^Neurons (*p* < 0.0001, Fig. 4B). The speed of anterograde movement of Lysostracker Red-positive vesicles was decreased 6-fold (*p* = 0.0003) from 1.63 ± 0.66 μm/s in control to 0.28 ± 0.44 um/s in NPC1 mutant i^3^Neurons (Fig. 4C). The distance that an acidic vesical traveled at one time (run length) was also significantly decreased in the anterograde direction, from 36.47 ± 17.14 to 5.31 ± 8.10 μm in *NPC1*^*−/−*^ neurons (*p* = 0.0062, Fig. 4D).

Treating *NPC1*^*−/−*^ i^3^Neurons with 100 μM HPβCD improved axonal lysosomal transport for all three parameters (Fig. 4B–D). The fraction of non-mobile acidic vesicles decreased significantly (*p* = 0.0007) from 81.4 ± 12.6 to 49.3 ± 5.7% in *NPC1*^*−/−*^ i^3^Neurons treated with HPβCD (Fig. 4B). Increased motility was observed in both anterograde (5 to 21%) and retrograde (14 to 30%) movements after treatment with HPβCD. Similarly, HPβCD increased both anterograde speed of movement from 0.28 to 0.94 μm/s (*p* = 0.4683) and run length from 5.31 to 27 μm (*p* = 0.2202) in *NPC1*^*−/−*^ i^3^Neurons. Videos of lysosomal movement in *NPC1*^*+/+*^ and *NPC1*^*−/−*^ axons with and without HPβCD treatment are provided in the Additional file 4: Supplemental video S1.


**Aditional file 4: Supplemental Video S1.** Videos showing axonal movement of acidic vesicles in NPC1+/+ and NPC1-/- i3Neurons with and without exposure to HPβCD. Kymographs generated from these videos are shown in Fig. 4.


## Discussion

In this study, we demonstrate that the NPC1 i^3^Neuron model offers a valuable tool to study cellular consequences of NPC1 dysfunction in neurons. Previous studies of NPC1 have utilized cell culture models including patient fibroblasts and iPSC-derived neuronal cultures [3, 22], as well as in vivo mouse and cat models [14, 23]. The *NPC1*^*−/−*^ i^3^Neuron model system has multiple advantages over standard iPSC-derived neurons. These advantages include the availability of an isogenic control, as well as rapid and simpler differentiation to a relatively homogeneous culture of glutamatergic neurons. These properties make the *NPC1*^*−/−*^ i^3^Neurons well suited for biochemical and cell biology studies as well as high-throughput chemical and molecular screens.

The *NPC1*^*−/−*^ i^3^Neurons accurately model endolysosomal storage of unesterified cholesterol and GM2. Although unesterified cholesterol accumulates in Lamp1^+^ vesicles, the total cellular content of unesterified cholesterol is not increased. This is consistent with prior work in NPC1 cell lines and the mouse models [14, 24, 25]. Thus, unesterified cholesterol storage is an issue of cellular distribution rather than absolute accumulation. Analysis by electron microscopy shows that the *NPC1*^*−/−*^ i^3^Neurons develop the multi-lamellar inclusion bodies that are characteristic of NPC1 disease. We also demonstrate that *NPC1*^*−/−*^ i^3^Neurons manifest both lysosomal and mitochondrial dysfunction and show that axonal acidic vesicle motility is impaired in the *NPC1*^*−/−*^ i^3^Neurons. HPβCD is a drug that has been shown to have therapeutic efficacy in mouse [5, 6] and cat [7] models of NPC1 [19] and potential efficacy in NPC1 patients [8]. In this paper, we show that HPβCD decreases unesterified cholesterol storage and improves both mitochondrial function and axonal transport of the acidic vesicles. Thus, this work provides a proof of principle that NPC1 mutant i^3^Neurons can be used to screen for potential therapeutic agents. A high-throughput drug screen in neurons may identify potential therapeutic compounds that would not be identified in fibroblast screens.

Neurons are highly polarized cells, and lysosomes located at the distal axon are thought to be immature, with retrograde transport needed to deliver lysosomes to soma to perform its degradative function [26], and such lysosome transport defects have been identified in a number of neurodegenerative diseases such as Alzheimer and Parkinson diseases [27]. Lysosomal movement has previously been measured at a speed of 1.91 μm/s in neurons [28], which is similar to what we observed for acidic vesicles in the *NPC1*^*+/+*^ i^3^Neurons (1.60 μm/s). However, the rate of lysosomal movement is significantly decreased 5-fold to 0.30 μm/s in the *NPC1*^*−/−*^ neurons and likely contributes to the perinuclear localization of PFO staining vesicles containing unesterified cholesterol.

Despite the lysosome being the immediate site of unesterified cholesterol and lipid accumulation, it is clear that there are also other cellular effects including mitochondrial dysfunction [29]. With deficiencies in both glycolysis and oxidative phosphorylation, the *NPC1*^*−/−*^ neurons do not match the energy production rates of the control *NPC1*^*+/+*^ neurons. Notably, the brain utilizes approximately 20% of total resting oxygen, with neurons possessing a disproportionately high demand for energy compared to other cell types in the body [30, 31]. Neurons rely on oxidative phosphorylation to meet this energy demand, whereas iPSCs and neural progenitor cells primarily utilize aerobic glycolysis [32]. Rapidly dividing iPSCs are more similar in their growth pattern to cancer cells, which rely on glycolysis to quickly generate ATP, a phenomenon known as the Warburg effect [33]. However, a transition toward oxidative phosphorylation during the neuronal differentiation process has previously been described [32]. This defect in mitochondrial function may contribute to the increased oxidative stress reported in NPC1 [34].

## Conclusions

This study highlights the value of an iPSC-derived neuronal model of NPC1 disease that is isogenic, recapitulates major NPC1 phenotypes, and can easily generate large numbers of homogenous neurons. In characterizing this cell line, we demonstrate the diversity of effects caused specifically by NPC1 deletion, which includes cholesterol accumulation at the lysosome, as well as broader changes to the lipid profile of the whole cell. In addition, we show defective lysosomal activity and transport, as well as downstream effects on mitochondrial function. This emphasizes the complexity of NPC1 disease, and the need to correct deficiencies beyond cholesterol accumulation alone. Thus, this cell line will provide a valuable tool for the NPC1 research community, in further studying the disease pathology and therapeutic discovery.

## Methods

### Cell culture and neuronal differentiation

Human iPSCs were cultured on Matrigel-coated dishes in Essential 8 Medium according to the manufacturer’s instructions. Briefly, cells were passaged routinely when 70–90% confluent with accutase and seeded into Essential 8 Medium supplemented with 10 nM Y-27632 dihydrochloride ROCK inhibitor. After 24–48 h, when iPSC colonies are visible, cells were refreshed with Essential 8 Medium and maintained until the next passage. Differentiation of i^3^Neurons was performed as described previously [9]. Briefly, iPSCs were seeded on day 0 into pre-differentiation medium (Knockout DMEM/F12 with 1× MEM non-essential amino acids, 1× GlutaMAX, 1× N2A supplement, 2 μg/mL doxycycline, and 10 nM Y-27632 dihydrochloride ROCK inhibitor). Cells were refreshed daily with a pre-differentiation medium on days 1 and 2 and split on day 3 with accutase. Cells were counted and re-seeded on poly-ornithine-coated dishes in neuronal medium (BrainPhys medium supplemented with 1× NeuroCult SM1, 10 ng/mL NT-3, 10 ng/mL BDNF, and 1 μg/mL mouse laminin). Every second day, half of the media was removed, and an equal volume of fresh media was added to maintain neuronal health.

### Generation of *NPC1*^*−/−*^ iPSC line

CRISPRi-i^3^Neurons iPSCs harboring a single-copy of doxycycline-inducible mouse neurogenin-2 (NGN2) at the AAVS1 locus was used as the parental line for genetic engineering. This parental line is a well-characterized control human iPSC line, WTC11. Multiple sgRNAs targeting *NPC1* were selected from the Brunello library [35] to maximize on-target activity and minimize off-target effects. sgRNAs were cloned into the lentiCRISPRv2 vector [36] containing *Streptococcus pyogenes* Cas9 nuclease and GFP; 3 μg DNA was transfected into iPSCs using Lipofectamine In-Stem, and after 3 days, GFP-positive cells were isolated by FACS sorting and genomic DNA extracted to perform the TIDE assay [37] to identify the effectiveness of each sgRNA in generating indels. The top two most effective guides that were less than 1 kb apart were used to generate the *NPC1*^*−/−*^ iPSC line. The two sgRNAs sequences are ACTGAACCTGTTTTGTGAGC and AAAGAGTTACAATACTACGT, which target exon 4 of *NPC1*. The two constructs were co-transfected (3 μg DNA total) into *NPC1*^*+/+*^ i^3^Neurons using Lipofectamine In-Stem, and after 3 days, GFP-positive cells were FACS-sorted. Individual clones were expanded from a single cell, and those with homozygous deletion of *NPC1* as determined by PCR genotyping were used for further testing. Karyotyping was normal for the clonal line (Figure S1A) used for further characterization and experiments in this study, which is referred to as the NPC1 i^3^Neuron line.

### Immunofluorescence

*NPC1*^*+/+*^ i^3^Neurons and *NPC1*^*−/−*^ i^3^Neurons were seeded at day 3 post-doxycycline on poly-ornithine-coated dishes compatible with microscopy (i.e., coverslips, Ibidi chambered slides, or Perkin-Elmer 96-well microplates) into the neuronal media. At the indicated time points, cultured cells were washed three times with 0.1 M PBS (pH 7.4) followed by fixation with 4% paraformaldehyde for 5 min and permeabilized with 0.05% Triton X-100. After blocking in 5% normal goat serum, the cells were stained with primary antibodies. For secondary antibodies, Alexa Fluor 488-, 564-, or 647-conjugated secondary antibodies (Thermo Scientific, Waltham, MA, USA) were used. To image cholesterol, i^3^Neurons were fixed with 4% paraformaldehyde and permeabilized with 0.1% Tween 20 in PBS prior to staining with the cholesterol marker perfringolysin-O conjugated to Alexa Fluor 488 in 0.1% Tween 20 with 3% donkey serum in PBS for 1 h. Hoechst 33342 (1 μg/mL, Invitrogen, Carlsbad, CA, USA) staining was used to visualize the cell nuclei. Primary antibodies included NeuN (Millipore, MAB377), β-tubulin (Abcam, ab18207), GluR1 (Santa Cruz, sc-7609), GluR2 (Santa Cruz, sc-7610), MAP2 (Millipore, MAB3418), GM2 ganglioside (Dr. Dobrenis, New York, USA), and LAMP1 (DSHB, H4A3). Cells were washed 3 times with PBS, stained with Hoechst nuclear stain for 5 min, and imaged using a spinning disk confocal microscope (Nikon Eclipse T*i*) controlled using Nikon Elements software.

### Measurement of cholesterol accumulation by microscopy

*NPC1*^*+/+*^ i^3^Neurons and *NPC1*^*−/−*^ i^3^Neurons were seeded at day 3 post-doxycycline on poly-ornithine-coated dishes compatible with microscopy (i.e., coverslips, Ibidi chambered slides, or Perkin-Elmer 96-well microplates) into the neuronal media. At day 10, cells were fixed with 4% paraformaldehyde for 15 min, permeabilized, and blocked with 0.2% Tween-20 + 3% donkey serum in PBS for 30 min. Cells were stained with 2 μg/mL perfringolysin-O (PFO) for 1 h and washed with PBS prior to imaging. Colocalization efficiency of PFO with Lamp1 was measured using the ImageJ software (http://rsb.info.nih.gov/ij/). The total numbers of puncta, intensity, and size were measured. For coverslips, Mowiol was used for mounting, and all imaging was performed with a spinning disk confocal microscope (Nikon Eclipse T*i*), controlled using the Nikon Elements software.

### Lipidomics

Fourteen days after doxycycline treatment, *NPC1*^*+/+*^ i^3^Neurons and *NPC1*^*−/−*^ i^3^Neurons were washed with PBS, and cell pellets were harvested for lipidomics analysis. Lipid levels were normalized to protein levels. Lipidomics were conducted as described by Fan et al. [38]. Full lipidomic data is available in Additional file 7.

### Measurement of lysosomal pH

Quantification of the lysosomal pH was performed as described previously [17], with minor modifications to allow automated, large-scale analysis. The fluorescent signal intensity of the Oregon-488 dextran is affected by changes in pH, enabling a standard curve to be generated by artificially adjusting extracellular pH, as well as measuring the pH of each cell line [17]. Briefly, *NPC1*^*+/+*^ i^3^Neurons and *NPC1*^*−/−*^ i^3^Neurons were seeded at day 3 on poly-ornithine-coated 96-well dishes compatible with microscopy into neuronal media. Half-media changes were performed every 2 days and on day 9, and neurons were co-loaded with 50 μg/mL pH-sensitive Oregon Green 488-dextran (Invitrogen, Carlsbad, CA, D7171) and 50 μg/mL pH-insensitive Alexa-Fluor-555 dextran (Invitrogen, Carlsbad, CA, D34679). After 4 h, cells were washed with PBS and chased overnight with fresh neuronal media to allow dextrans to accumulate in the late endosomal/lysosomal compartment. To develop the calibration curve, *NPC1*^*+/*+^ i^3^Neurons were incubated for 10 min in physiological buffer solutions of varying pH (3–8) in the presence of 10 μg/mL nigericin to force intracellular pH to equilibrate with the surrounding environment. The ratio of the sum intensity of green to red fluorescence measured and Oregon Green 488 fluorescence was inversely related to pH. *NPC1*^*+/+*^ i^3^Neurons and *NPC1*^*−/−*^ i^3^Neurons were imaged and analyzed using the Opera Phenix High Content Screening System (Perkin Elmer) and plotted against the calibration curve to determine pH.

### Electron microscopy

Day 3 i^3^Neurons were seeded on glass coverslips and fixed at day 21 in 2.5% glutaraldehyde made in 0.1 M sodium cacodylate buffer, pH 7.4. The following processing steps were carried out using the variable wattage Pelco BioWave Pro microwave oven (Ted Pella, Inc., Redding, CA.): cells were rinsed in 0.1 M sodium cacodylate buffer, pH 7.4, post-fixed in 1% osmium tetroxide made in 0.1 M sodium cacodylate buffer, rinsed in double-distilled water (DDW), 3% (aq.) uranyl acetate enhancement, DDW rinse, and ethanol dehydration series up to 100% ethanol, followed by an Embed-812 resin (Electron Microscopy Sciences, Hatfield, PA.) infiltration series up to 100% resin. The epoxy resin was polymerized for 20 h in an oven set at 60 °C. Ultra-thin sections (90 nm) were prepared on a Leica EM UC7 ultramicrotome. Ultra-thin sections were picked up and placed on 200-mesh copper grids (Electron Microscopy Sciences, Hatfield, PA) and post-stained with uranyl acetate and lead citrate. Imaging was performed on a JEOL-1400 Transmission Electron Microscope operating at 80 kV, and images were acquired on an AMT BioSprint 29 camera.

### Oxygen consumption rate measurements

Oxygen consumption rate (OCR) was measured in i^3^Neurons using the XF96 Extracellular Flux analyzer (Seahorse Bioscience) following the manufacturer’s protocol. In short, day 3 i^3^Neurons were seeded at 2 × 10^4^ cells per well in XF96 cell culture multi-well plates in neuronal medium and incubated for 4 days in the differentiation conditions stated for i^3^Neurons cultures. Prior to the last day of culture, XF96 cartridges were incubated overnight in XF calibrant at 37 °C in a non-CO_2_ incubator. The neuronal medium was exchanged with artificial cerebrospinal fluid incubated at 37 °C in a non-CO_2_ incubator for 1 h before OCR measurement. Inhibitors provided in the kit were diluted to suggested concentrations in artificial cerebrospinal fluid and loaded into corresponding microwells in the XF96 cartridge plate. After equilibration of sensor cartridges, the XF96 culture plate was loaded into the XF96 Extracellular Flux analyzer for OCR measurements.

Basal OCR and ECAR were determined by measuring the mitochondrial stress test profiles with sequential injection of oligomycin (O), FCCP (carbonyl cyanide-p-trifluoromethoxyphenylhydrazone) (F), and rotenone and antimycin A combination (A). Oligomycin inhibits the ATP synthase (complex V) reducing electron flow. Oligomycin was injected to a final concentration of 1 μM. FCCP is an uncoupling agent of oxidative phosphorylation and acts to depolarize the mitochondrial membrane potential; FCCP was injected to a final concentration of 0.5 μM. Rotenone/antimycin A mixture is a complex III inhibitor and was injected at a final concentration of 0.5 μM rotenone /0.5 μM antimycin A. By subtracting non-mitochondrial respiration from the basal rate prior to oligomycin injection, basal respiration was calculated. Maximal respiration was calculated by subtracting the rate after injection of FCCP with non-mitochondrial respiration. Rotenone/antimycin A was injected to measure non-mitochondrial respiration.

### Microfluidic assays

Polydimethylsiloxane (PDMS) microdevices with two open chambers connected by microgrooves 5 μm wide, 4.5 μm high, and 450 μm long were molded on photolithographically patterned templates designed and fabricated in-house, and then the devices were irreversibly bonded to acid-etched glass coverslips after activation with oxygen plasma. The devices were coated with poly-l-ornithine for 1 h. Cells were differentiated for 3 days then plated in one chamber of the microdevice and cultured for 4 days to allow the axons to grow through the microgrooves to the other chamber for easy visualization. After 4 days in culture, the medium was supplemented with 100 μM hydroxypropyl-β-cyclodextrin (HPβCD) (+) or left untreated (−). LysoTracker Red was then added to detect lysosomes, and the axon compartment was imaged. One hundred-micrometer segments of the axon were sequentially recorded every 0.2 s for 5 min. Kymographs were generated with Fiji: lines of one-pixel thickness and 100-μm length were tracked through the axon and straightened, followed by stack re-slicing and Z-projection. The number of events, speed, and run length was determined from kymographs with Fiji ROI manager. Particles with negative and positive slopes represent the vesicles moving in the anterograde and retrograde directions, respectively. Live-cell imaging was performed on a spinning-disk Eclipse T*i* Microscope System (Nikon). Cells were kept at 37 °C and 5% CO_2_, within a humidified environmental chamber. Confocal images were taken with a × 60 objective (N.A. 1.40) Plan Apo VC and a high-speed electron-multiplying charge-coupled camera (iXon Life 897; Andor). Acquisition was performed with the NIS-Elements AR microscope imaging software.

### Statistical analysis

The results are expressed as mean ± SD of the number of independent experiments. All experiment was reproduced at least three times on different days. Comparison between the two groups was performed by the non-parametric Mann-Whitney test, and comparison between multiple groups was performed by non-parametric one-way analysis of variance (ANOVA). Statistical analyses were performed using GraphPad Prism version 8.0 (GraphPad Software, San Diego, CA, USA)

## Supplementary Information

**Additional file 1: Figure S1.** i^3^Neurons and inducible differentiation. **(A)** Schematic showing integrated doxycycline-inducible neurogenin-2 (NGN2) transcription factor in the AAVS1 locus of the i^3^Neuron iPSC line. **(B)** The i3Neuron iPSCs are initially grown in E8 medium. The iPSCs are then moved to N2 pre-differentiation medium and differentiation was induced by addition of doxycycline (Dox) to the medium (N2(Dox)). Three days after initiation of differentiation the cells are replated in neuronal medium. Neurons are fully differentiated by day 10 **(C)** Brightfield images of *NPC1*^*+/+*^ i^3^Neurons as iPSCs and over the neuronal differentiation process. **(D)** Images of day 10 control i^3^Neurons stained with NeuN (neuronal nuclei; green) and Hoechst (nuclei; blue) Scale bar, 10 μm.

**Additional file 2: Figure S2. (A)** Karyotyping of monoclonal NPC1^-/-^ i^3^Neuron cell line shows a normal male karyotype. **(B)** *NPC1*^*+/+*^ i^3^Neurons and *NPC1*^*-/-*^ i^3^Neurons were differentiated for 10 days, fixed and stained with β-tubulin, MAP2, GluR1 and GluR2. Nuclei were counter stained with Hoechst (blue). Data are from at least 100 cells per condition; Images are representative of three independent experiments Scale bar, 50 μm.

**Additional file 3: Figure S3.** Comparison of cyclodextrins on NPC1 i^3^Neuron cholesterol accumulation. *NPC1*^*+/+*^ i^3^Neurons and *NPC1*^*-/-*^ i^3^Neurons were differentiated for 10 days and treated with or without the indicated concentrations of hydroxypropyl-β-cyclodextrin (CD), sulfobutylether-β-cyclodextrin, or sulfobutylether-γ-cyclodextrin for 24 h. Cells were then fixed and stained with PFO-488 which stains unesterified cholesterol and Hoechst nuclear stain. Images were analyzed for PFO-488 signal intensity and these data were normalized to nuclear count. Data are representative of one experiment, with six replicate wells per treatment condition.

**Additional file 5.** Raw-data-Numerical.

**Additional file 6.** Raw-data-Western blotting.

**Additional file 7.** Raw-data-Lipidomics in i3Neuron Full.

## Data Availability

All data generated or analyzed in this study are included in this published article and the accompanying supplementary information files. All raw data used in the generation of the figures can be found in Additional files 5 and 6. Full lipidomic data is available in Additional file 7.

## Abbreviations

NPC: Niemann-Pick disease
NPC1: Niemann-Pick disease, type C1
iPSC: Induced pluripotent cell
HPβCD (CD): 2-Hydroxypropyl-β-cyclodextrin
OCR: Oxygen consumption rate
ECAR: Extracellular acidification rate
FCCP: Carbonyl cyanide-p-trifluoromethoxyphenylhydrazone
Dox: Doxycycline
