# Supplementary figures and images for: A human iPSC-derived inducible neuronal model of Niemann-Pick disease, type C1
Source: BMC Biol. 2021 Oct 1;19:218. doi: 10.1186/s12915-021-01133-x (PMC8485536; doi:10.1186/s12915-021-01133-x)

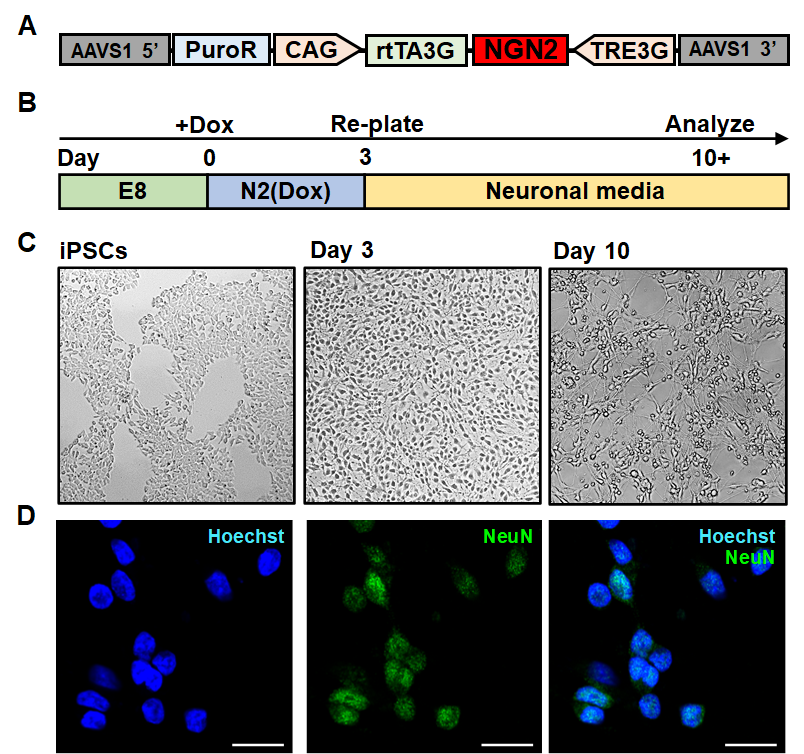

Supplement: Supplementary file 2 — Additional file 1: Figure S1. i3Neurons and inducible differentiation. (A) Schematic showing integrated doxycycline-inducible neurogenin-2 (NGN2) transcription factor in the AAVS1 locus of the i3Neuron iPSC line. (B) The i3Neuron iPSCs are initially grown in E8 medium. The iPSCs are then moved to N2 pre-differentiation medium and differentiation was induced by addition of doxycycline (Dox) to the medium (N2(Dox)). Three days after initiation of differentiation the cells are replated in neuronal medium. Neurons are fully differentiated by day 10 (C) Brightfield images of NPC1+/+ i3Neurons as iPSCs and over the neuronal differentiation process. (D) Images of day 10 control i3Neurons stained with NeuN (neuronal nuclei; green) and Hoechst (nuclei; blue) Scale bar, 10 μm. [file 12915_2021_1133_MOESM1_ESM.tif]

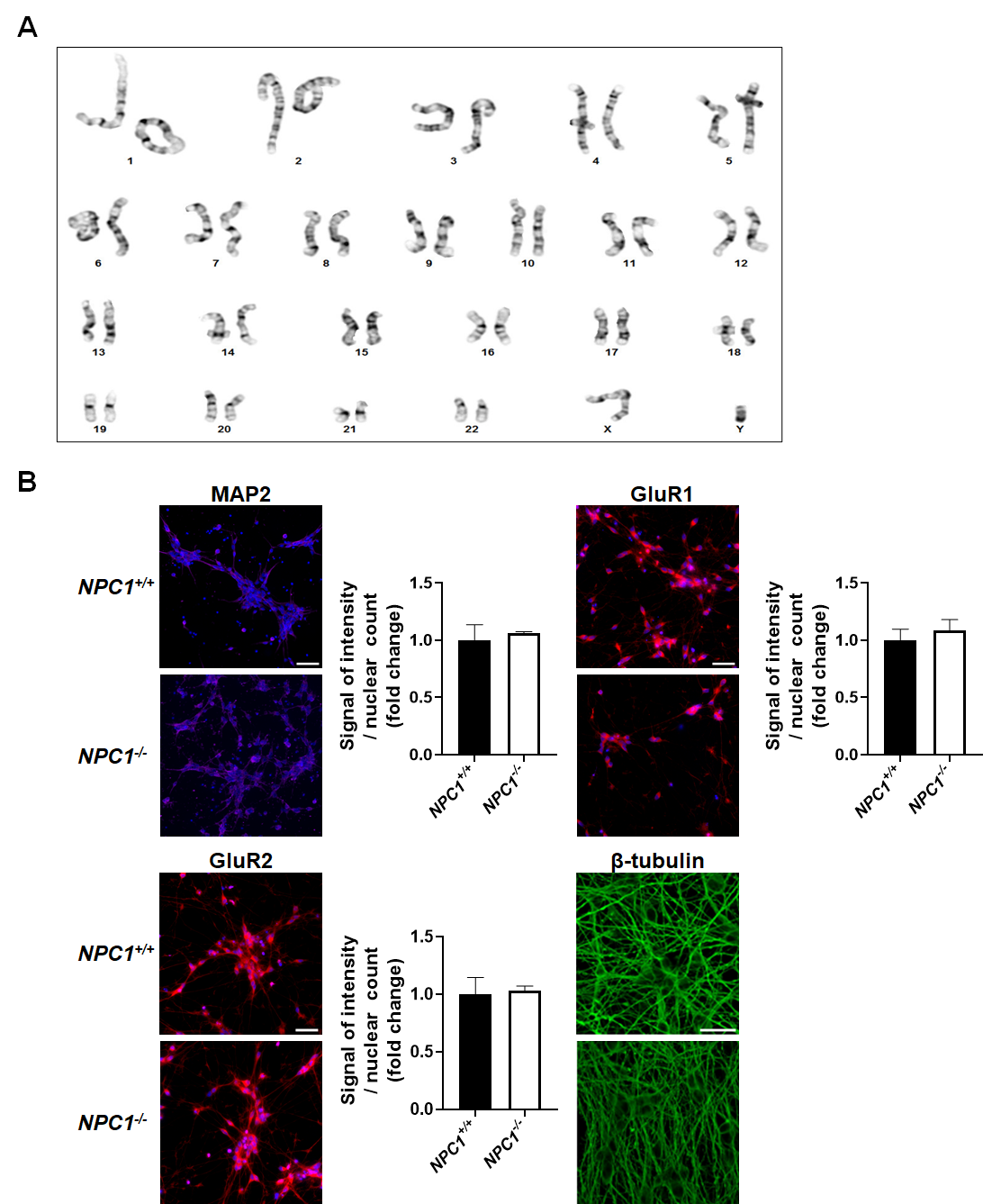

Supplement: Supplementary file 3 — Additional file 2: Figure S2. (A) Karyotyping of monoclonal NPC1-/- i3Neuron cell line shows a normal male karyotype. (B) NPC1+/+ i3Neurons and NPC1-/- i3Neurons were differentiated for 10 days, fixed and stained with β-tubulin, MAP2, GluR1 and GluR2. Nuclei were counter stained with Hoechst (blue). Data are from at least 100 cells per condition; Images are representative of three independent experiments Scale bar, 50 μm. [file 12915_2021_1133_MOESM2_ESM.tif]

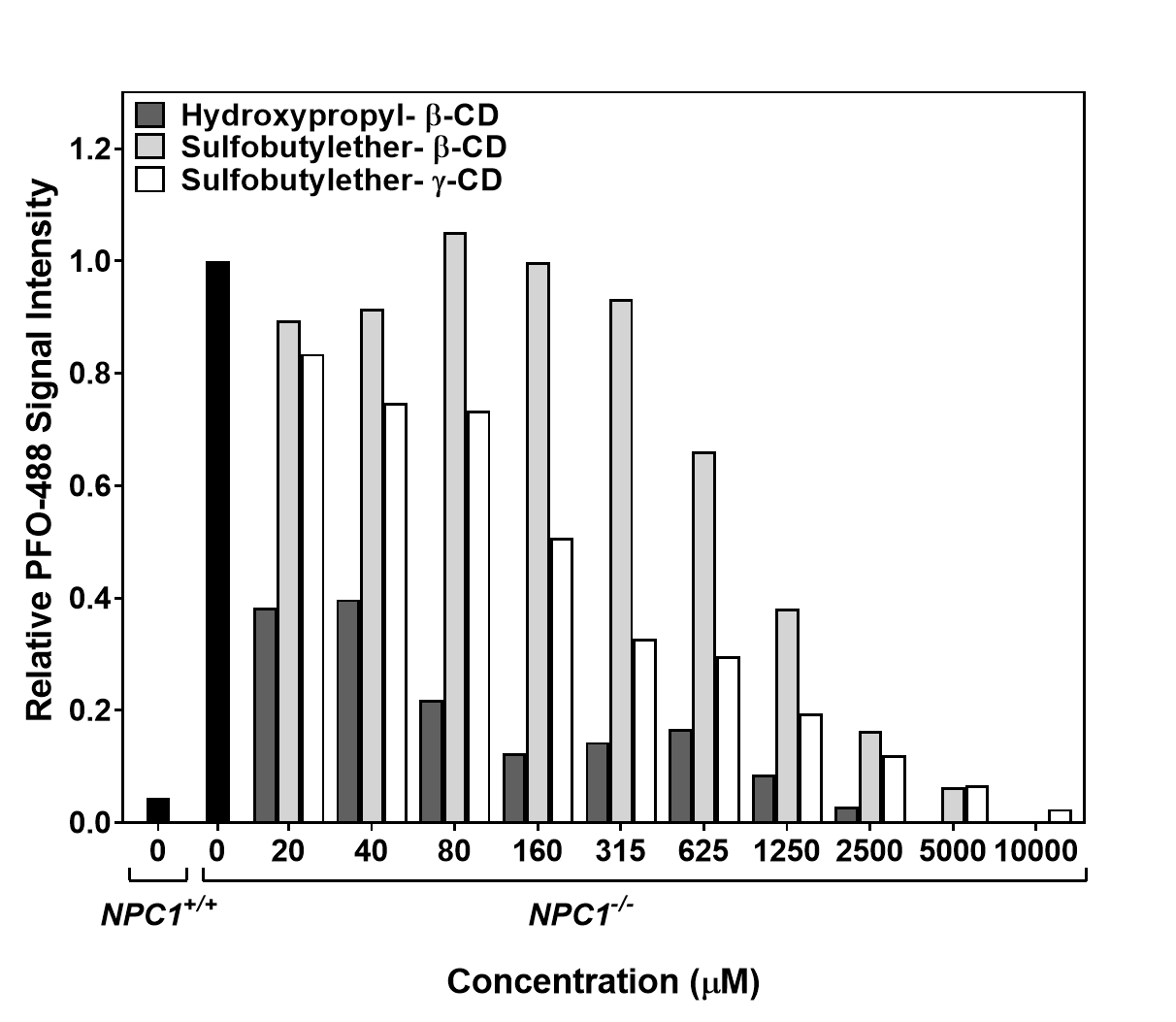

Supplement: Supplementary file 4 — Additional file 3: Figure S3. Comparison of cyclodextrins on NPC1 i3Neuron cholesterol accumulation. NPC1+/+ i3Neurons and NPC1-/- i3Neurons were differentiated for 10 days and treated with or without the indicated concentrations of hydroxypropyl-β-cyclodextrin (CD), sulfobutylether-β-cyclodextrin, or sulfobutylether-γ-cyclodextrin for 24 h. Cells were then fixed and stained with PFO-488 which stains unesterified cholesterol and Hoechst nuclear stain. Images were analyzed for PFO-488 signal intensity and these data were normalized to nuclear count. Data are representative of one experiment, with six replicate wells per treatment condition. [file 12915_2021_1133_MOESM3_ESM.tif]

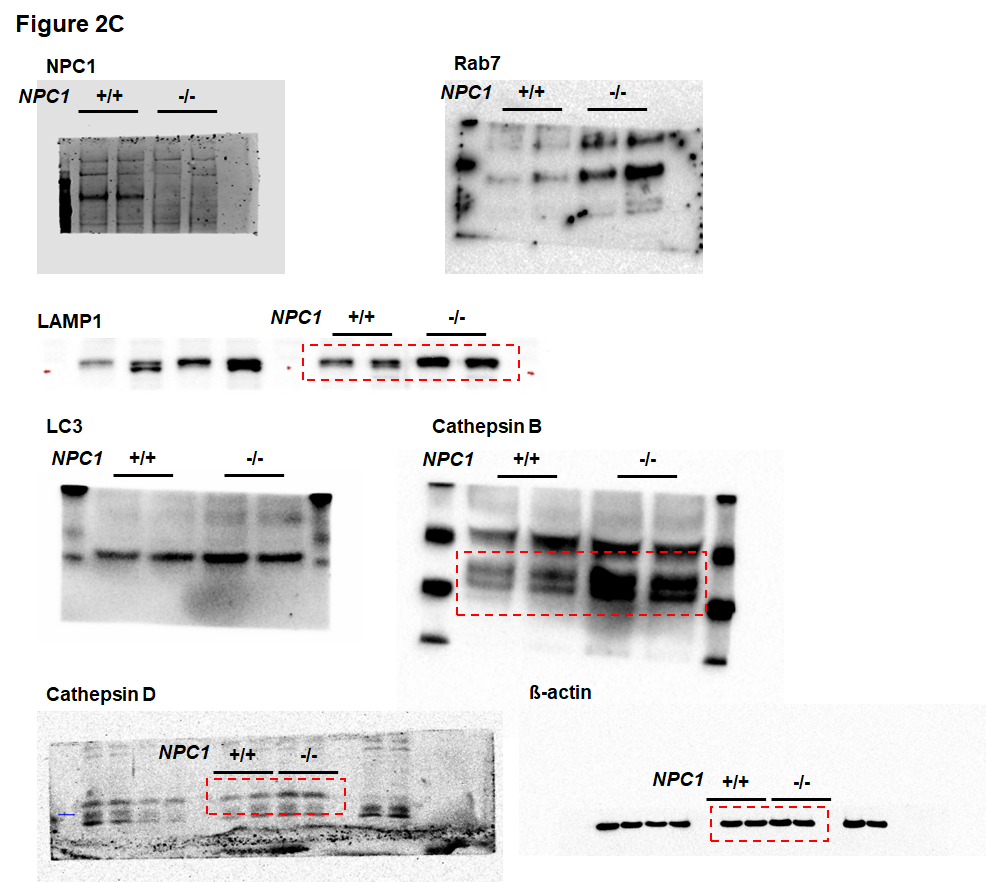

Supplement: Supplementary file 6 — Additional file 6. Raw-data-Western blotting. [file 12915_2021_1133_MOESM6_ESM.tif]
